# A Bourdieusian Approach to the Demobilisation of Brazil's AIDS Movement

**DOI:** 10.1111/1467-9566.70187

**Published:** 2026-04-22

**Authors:** Helena de Moraes Achcar

**Affiliations:** ^1^ São Paulo School of Administration Getúlio Vargas Foundation São Paulo Brazil

## Abstract

This article offers a Bourdieusian analysis of the demobilization of Brazil's AIDS movement, once a globally celebrated force in shaping innovative, rights‐based public health responses. Drawing on extensive qualitative data, I argue that the movement's decline cannot be explained solely by institutional co‐optation, biomedicalisation, or defunding, as suggested in previous scholarship. Instead, I conceptualise demobilisation as a deeper process of depoliticisation that emerges from transformations in the distribution of capital, shifts in habitus, and structural changes in the field of power under neoliberalism. I trace how the pauperisation of the epidemic brought into the field new agents with different dispositions, challenging the movement's politicised doxa forged in the 1980s and 1990s and fragmenting the field. The analysis emphasises the role of symbolic struggles over legitimacy and the effects of changing political‐economic logics on activist practices. By combining field theory with a grounded empirical study of Brazil's AIDS movement, the article contributes to broader debates on the sociology of social movements, the neoliberal restructuring of civil society and the conditions of possibility for re‐politicisation.

## 1 Introduction

Brazil's civil society played a critical role in the country's response to AIDS. Thanks to a politically active movement the country became the first in the developing world to provide free treatment to all people living with HIV (PLHIV), earning international recognition for its groundbreaking AIDS programme (see Parker 2003; Grangeiro et al. 2009). Born in the mid 1980s, during re‐democratisation, Brazil's AIDS movement was able to present a radical challenge to the state's initial approach (or lack thereof) to AIDS and to the socio‐political status quo that characterised the country under a military dictatorship. Under democratic governance, through a ‘state‐movement’ alliance (Rich 2019), it managed to shape policy and participate in important decisions, such as the state's compulsory licencing of efavirenz in 2007 (Nunn et al. 2012; Grangeiro et al. 2009).

However, since the late 2000s, the movement has been described by activists as ‘weak’ and ‘incapable of fighting as before’ (see Landau 2011). Despite great past achievements, reasons for mobilisation abound, according to activists: lack of funds for work with prevention, ineffective approaches to treatment (see Grangeiro 2016), and the prevalence or growth of HIV in certain subgroups (Ministério da Saúde 2023). This article explores how and why such a politicised institutionally integrated movement lost its public and political force.

Like the broader literature on social movements (see Michels 1949 and Collier and Collier 1991 in Rich 2019; see also della Porta and Diani 2020; Lapegna 2013), the literature on Brazil's AIDS movement argues that it is precisely institutionalisation, state support and resources that have co‐opted activists and contributed to the decline of the movement (Paiva 2003; Parker 2003). Others have focused on the defunding of NGOs, particularly in the late 2010s, by international donors and the state (Parker 2011; Parker et al. 2022; Fonseca 2007), or the 2020 far‐right government attack on human rights and closure of spaces for social participation (Malta and Beyrer 2013; Greco 2016) to explain demobilisation. Studies have also explored the effect of the ‘pharmaceuticalisation’ or ‘re‐biomedicalisation’ of AIDS policy (see Biehl 2007) and the simultaneous emergence of the ‘end of AIDS’ discourse (Kenworthy et al. 2017), which have reduced AIDS to a biomedical issue and masked structural inequalities which many of the policies and the movement had sought to address (see Kenworthy et al. 2017). From a Bourdieusian perspective, Barros and Silva (2016) and Barros (2018) contend that the increasing technical (and medical) specialisation and complexity of Brazil's AIDS space in the late 1980s and 1990s led to the proliferation of various commissions. This institutional fragmentation ultimately diluted the influence and political force of NGOs in the AIDS space.

However, important facets of the process of demobilisation remain underexplored. First, the movement's own internal dynamic, that is, the relationship of competition between organisations and actors who fight for the same goals. Second, the symbolic dimension of this competition, that is, the struggle to define what legitimate anti‐AIDS activism should be. As the next section explains, Bourdieu's approach to sociology (1977) and its main concepts of field (an objective and bounded space of struggle and competition), habitus (actors' embodied dispositions), capital (resources) and the related concept of doxa (the dominant and naturalised discourse) allow me to capture the symbolic and internal dynamics of the field and their interaction with changes in external environments.

I argue that the AIDS movement not only became demobilised, that is, its collective actions and contentious claims decreased (Tilly and Tarrow 2015), but was, in fact, depoliticised. The broader literature on social movements treats depoliticisation as a process that involves the loss of contentious politics, radical ideology, and autonomous action, typically through institutional co‐optation or state intervention and repression (Thörn and Svenberg 2016; Uitermark and Nicholls 2014; Davenport 2014). Athough these definitions of depoliticisation may also be true for Brazil's AIDS movement, I offer a relational, field‐theoretical analysis that links structural transformation, symbolic capital, and habitus to explain depoliticisation as a deeper sociological process rather than simply the result of co‐optation or institutionalisation.

The field of the AIDS movement is, like all fields, unequal and hierarchical. In the 1980s and 1990s, dominant NGOs possessed a combination of cultural (formal education), social (connections with—and access to—politicians and policymakers) and economic capital (funds from foundations and the state), which gave them symbolic power (legitimacy and recognition) and allowed them to set the rules of the game (Bourdieu 1998a), that is, the underlying principles that should guide organisations' activism. The doxa of the field incorporated principles such as human rights, democracy and social determinants of health, all linked with solidarity as the key principle in the fight against AIDS. These were part of a broader political project put forward by actors who possessed the adequate habitus acquired through their militant and political history.

I trace the roots of the movement's depoliticisation to the effects of the epidemic's pauperisation, which brought different and urgent needs to the fight against AIDS, as well as new activists with different habitus, competencies and capital. Simultaneous changes in the international development field, the Brazilian AIDS policy field and more broadly the field of power help account for the fragmentation and the challenge to the politicised doxa of the 1980s and 1990s.

In accounting for the process of depoliticisation, a Bourdieusian approach allows me to explore the conditions of possibility for political action not only in terms of wider circumstances but also in terms of positions actors occupy in the field based on the amount and combination of resources they own, and on their dispositions, which are historically acquired. Actors with certain dispositions will act when there are social conditions for it, in this case social, economic and militant capital.

### 1.1 A Bourdieusian Approach to the Study of the AIDS Movement

To understand the AIDS movement as a social field in the Bourdieusian sense is to understand it as a relatively structured social space, which consists of positions occupied by agents and the configuration of power relations between them (in this case AIDS NGOs or networks and whether they dominate the field or not), and the interests, rules and principles that govern these relationships (Bourdieu 1984b; Thomson 2012). A field operates as a battlefield ‘in which various actors struggle for [its] transformation or preservation […]’(Bourdieu 1998a, 40), and their actions produce effects upon each other. ^1^

At stake is the accumulation of capital, not only economic but also cultural (e.g., education) and social capital, defined as networks of ‘more or less institutionalised relationships of mutual acquaintance and recognition’ (Bourdieu and Wacquant 1992, 119). The strategies actors employ are shaped by the capital they hold (Husu 2013). Capitals vary in value across fields and when relevant to a specific field, they become symbolic capital, that is, imbued with status, recognition and authority. In the AIDS movement, dominant NGOs born in the 1980s and 1990s were staffed with people who possessed cultural and social capital which ultimately had symbolic value. As part of the educated middle class with university degrees mostly in humanities, social sciences, medicine or other health‐related area, and with a trajectory of political activism in the fight for democracy, activists managed to attract economic resources from donors who valued their education and were sympathetic towards their political history and fight (Interview 5, 7, 8 and 26). Activists also had access to policymakers, particularly the *sanitaristas* who in many instances led the AIDS division within health secretariats and shared a common political trajectory in the broader cycle of social movements that unfolded in the 1980s (see Parker 2003). This configuration of capital accrued by these political NGOs afforded them the possibility to establish what was legitimate in terms of AIDS activism, that is, to set the rules of the game.

Although fields are hierarchical, Bourdieu argued they can change, especially when influenced by external events (Thomson 2012). For example, I show how the World Bank's loans promoted the technicisation of NGOs and encouraged a movement of PLHIV to draw on different types of symbolic capital (their lived experience of HIV) which conferred legitimacy, especially over activists without the virus. This shift coincided with the epidemic's pauperisation in the 1990s. Although initially a political challenge to the field's power dynamics, these new strategies—and the resulting fragmentation—eventually contributed to a depoliticising effect on the activist doxa, reflecting broader transformations in the field of power.

Actors' strategies are also shaped by their habitus. Bourdieu (1977), defines habitus as ‘a subjective but not individual system of internalised structures, schemes of perception, conception, and action common to all members of the same group or class’. These structures inform how agents perceive and act upon the epidemic. The politicised habitus of 1980s activists reflected their backgrounds in formal education and political training. In contrast, the pauperisation of the epidemic brought in activists with different habitus (often lacking these resources), who contested the principles of earlier activism, such as solidarity, and sought to alter the balance of power. Moreover, the material conditions of the field had shifted, and no longer matched the dispositions of the 1980s habitus. The new, urgent needs demanded a different logic and practice of activism.

For Bourdieu, habitus is both a generative structure and a product of past conditions. Section four will explore how the habitus of dominant activists was shaped by their history of struggle for democracy and human rights. This perspective helps illuminate the conditions that formed their politicised habitus and offers insight into the possibilities for re‐mobilising or re‐politicising the AIDS struggle.

### 1.2 Research Methods

This study received ethical approval from the Fundação Getúlio Vargas Ethics Committee. For the analysis, I conducted 41 in‐depth semi‐structured interviews in 2023 and 2024. Twenty were with activists affiliated with major NGOs founded in the 1980s and 1990s—mostly in Rio, São Paulo, and Porto Alegre. All were still active today, though three now work as consultants for the Ministry of Health or other organisations. Some also belonged to networks of PLHIV (less formal or professionalised organisational models) but identified primarily as NGO activists or consultants. Seven interviewees were activists within networks founded in the 1990s and 2000s. The remaining were former or current Ministry of Health staff, consultants with AIDS‐related organisations and scholars whose research and involvement focused on HIV/AIDS. Following a Bourdieusian methodology, the goal was not to produce a statistical sample but to purposively or snowball‐select individuals who held influential positions in the field (Hardy 2012), shaping field‐specific capital.

Interview data was complemented by document analysis (from the Ministry of Health, NGOs, networks, and newspapers) and participant observation at support groups and activist events in Parliament. I focused on actors' backgrounds and socialisation—social, political and professional trajectories—and how these shaped their knowledge, skills and engagement in the AIDS struggle. Mapping actors' habitus and the distribution of capital helped identify their positions and the objective structure of relations in the field (Hardy 2012). Key NGO activists from the 1980s and 1990s had high education levels (eight PhDs, 12 university degrees), mostly from middle‐ or upper–middle‐class families, often involved in earlier political struggles such as re‐democratisation or minority rights. In contrast, most activists in PLHIV networks had different trajectories. Three had degrees in social sciences or humanities, whereas four had limited formal education due to lack of opportunity. Their activism usually began after their HIV diagnosis, within these networks.

I analysed transcripts, documents, and secondary literature for recurring themes and discourses, particularly regarding the principles and practices shaping AIDS activism. Following Bourdieu's critique of ‘objectivist knowledge’ (Bourdieu 1977), I do not separate data and results but instead weave them into the analysis that follows, tracing the field's origins and subsequent transformations.

## 2 The Genealogy of Activists' Dispositions and the Construction of a Political Doxa

The emergence of the AIDS epidemic in Brazil coincided with a historical moment that was marked by profound political transformations. The 1980s saw Brazil go through a process of re‐democratisation which eventually ended 21 years of military dictatorship. With the opening of the political regime in the late 1970s (*abertura*, in Portuguese) and the return of political exiles from abroad, several NGOs were founded and staffed by opponents of the military rule, typically from the educated middle class, who were committed to opposition politics and human rights (Parker 2003). What started as a response led by activists from gay organisations soon benefitted from the rapid expansion of the so‐called ‘NGO movement’, which created a fertile space for the emergence of the AIDS movement (Parker 1997, 2003). In 1985, the first AIDS NGO, the AIDS Prevention and Support Group (Portuguese acronym GAPA) was established in São Paulo (Valle 2002).

In Rio, two of the main and first AIDS NGOs were founded by leading figures of the NGO movement and political activists who had returned from exile. These were activist and sociologist Herbert de Souza (known as Betinho) and political activist Herbert Daniel who both lived with HIV and founded the Brazilian Interdisciplinary AIDS Association (Portuguese acronym ABIA). Daniel later founded the *Grupo pela Valorização, Integração e Dignidade do Doente de Aids* (Pela Vidda) and was also one of the founders of the Brazilian Green Party (Parker 2003). Although studying medicine, Daniel became involved in political activism as the vice‐president of the university students' union before joining the armed struggle against the military dictatorship (Green 2018). His writings and activism addressed the intersection of stigma, sexuality, and illness (Green 2018).

Betinho, trained in sociology and public administration, was widely known as a champion of human rights and an outspoken advocate against poverty. In addition to co‐founding ABIA, he established the Brazilian Institute of Social and Economic Analysis (Ibase), which played a major role in promoting democratic participation (Parker 2003). Both Daniel and Betinho were the ‘galvanizing agents’ of the mobilisation against AIDS in Brazil (Camargo Júnior 2003) and, alongside other AIDS activists, possessed what Crossley (2003) called ‘radical habitus’, developed by taking part in major political events—or ‘militant capital’, that is, knowledge and know‐how that could be mobilised in collective actions (Matonti and Poupeau 2004).

More broadly, these activists framed the struggle against AIDS as inseparable from the broader fight for human rights and democracy. They believed that an effective response to the epidemic required a democratic political field—one that allowed for participation, rights recognition and the inclusion of historically marginalised groups, such as homosexuals, women, and trans people (Interview 1). In this sense, important policy achievements such as universal access to antiretroviral (ARV) treatment were seen not only as technical responses to a public health crisis but as symbolic victories toward a more just and democratic social order (Parker 2020). This framing was central to the movement's ability to attract international funding. As one activist explained, ‘[f]unders liked having [in their recipient list] these activists who also participated in the re‐democratisation of the country—it gave them legitimacy’ (Interview 26). The accumulated symbolic capital of these actors—derived from their previous struggles, intellectual recognition and moral authority—translated into access to external resources. Their political biographies, media visibility, and connections with policymakers positioned them advantageously within the AIDS movement field.

Crucially, the Brazilian AIDS movement did not emerge in isolation. It was strongly influenced by the *sanitarista* movement, a progressive alliance of doctors, public health professionals, academics and activists who had, since the 1950s, called for a universal and participatory public health system. Their vision, rooted in principles, such as equity, universality and the right to health, was institutionalised in the creation of the *Sistema Único de Saúde* (SUS), Brazil's universal healthcare system, and enshrined in the 1988 Constitution (Grangeiro et al. 2009). These principles also informed the AIDS movement's orientation, reinforcing a conception of health as a social right.

Moreover, the *sanitaristas* challenged the dominant biomedical paradigm by foregrounding the social determinants of health, that is, the structural conditions (poverty, inequality, race, gender and labour precarity) that shape vulnerability to disease (Buss and Pellegrini Filho 2007; Parker et al. 2022). AIDS activists and sanitaristas developed close ties, with several figures from the health reform movement occupying key state positions. The first federal AIDS coordinator, Dr. Lair Guerra, was herself a sanitarista whose government created CNAIDS (the National AIDS Commission), a participatory body that institutionalised civil society's involvement in AIDS policymaking (Barros and Silva 2016).

This alliance illustrates the extent to which AIDS activists came to hold and deploy social capital, enabling them to influence the state from within while maintaining a position of relative autonomy (see Rich 2019). Their access to the media—another key field—further amplified their symbolic capital (Parker 2003, 2020; Nunn et al. 2012). As Bourdieu (2005) observed, the journalistic field plays a critical role in bestowing visibility and symbolic legitimacy across other fields. In the 1980s and 1990s, media exposure reinforced the activists' moral authority, contributing to the consolidation of their position within the AIDS movement and policy fields.

A recurrent theme in interviews, secondary literature and archival NGO materials from the 1980s and 1990s, was the centrality of solidarity as a principle underpinning activism. Solidarity was not conceived as charity, but as a political force grounded in horizontality and collective struggle. Political NGOs saw their mission as supporting the demands of oppressed or excluded groups, acting as mediators between grassroots movements and institutional arenas (Landim 1988; Oliveira Neto 1991 in Landim 1993).

AIDS NGOs were thus conceived as political actors, not service providers. Unlike technocratic or neoliberal logics that would later dominate parts of civil society (Lang and et al. 1997; Roy 2014), these organisations rejected the idea of civil society as a substitute for the state. Rather, they sought to pressure the state to fulfil its obligations, particularly with respect to prevention and treatment (Interviews 1, 43, 47). As one activist explained, solidarity was premised on a horizontal relationship between equals, in contrast to the verticality of donor–recipient dynamics that characterise charity (Interview 45; Roth 2019).

This politicised conception of solidarity was articulated in Daniel's (1994) *Vida Antes da Morte*, where he described it as ‘the only political force capable of transforming the world and fighting the disease’. For Daniel, solidarity was both ethical and political: it demanded the transformation of a society structured by exclusion and the recognition of diversity as a precondition for health. He advanced a universalist conception of PLHIV—a category that extended beyond seropositivity to include all those whose lives were affected by the epidemic (Terto 1999; Valle 2002). This symbolic strategy enabled a broader base of mobilisation, one that transcended individual diagnosis (or what Valle referred to as ‘clinical identity’) and produced a collective political subject: ‘the whole of humankind lives with AIDS’ (Terto 1999, 333). This conception structured the field of AIDS activism, shaping the ethos of NGOs such as Pela Vidda, which explicitly encouraged both HIV‐positive and HIV‐negative individuals to assume leadership roles (Terto 1999; Valle 2002). Although conflicts over resources and symbolic boundaries between ‘seropositives’ and ‘seronegatives’ would later challenge this conception within NGOs like Pela Vidda, until the first half of the 1990s universalism would provide the main framework for collective action in the field (Valle 2002).

Betinho had introduced a similar understanding of solidarity, linking it to respect for diversity and the idea that the AIDS response must be collective, not individualised (Parker 2014). As Seffner and Parker (2016), argue, to exercise solidarity ‘in its full political sense’ was to denounce a political order that rendered some lives more precarious than others. Solidarity functioned as a rallying principle, reinforcing the idea that the struggle against AIDS was connected to broader contests over inequality, social justice and the structural conditions that underpinned the epidemic (Parker et al. 2022).

Although some leading political NGOs engaged in activities that might be classified as charity—such as establishing hospices for PLHIV (see Contrera 2000)—they remained fundamentally committed to advocacy and the production of critical consciousness. Their central role was not the provision of services, but the mobilisation of symbolic capital to pressure the state and society. Acting as critical agents, these NGOs sought to reframe AIDS as a question of citizenship and rights rather than pathology or morality. Through public education campaigns, workshops, and training sessions, organisations such as ABIA worked to educate the population—especially in the peripheries of Rio de Janeiro and other major cities—and shape the interpretive frameworks through which AIDS was understood (Interviews 1, 43; Parker 2003).

Thus, these organisations did not merely respond to the epidemic; they produced the conditions for political engagement, setting the symbolic and cognitive structures of the field. Their research, policy analysis and media interventions functioned as mechanisms of field construction, defining the legitimate language, actors, and modes of participation in the AIDS policy arena (see Parker 2003; Interview 26). Therefore, they played a foundational role in structuring the habitus of newer activists who entered the field in other regions of Brazil. The principles, dispositions, and interpretive schemas developed by these pioneering NGOs became generative structures that oriented perception and practice for subsequent generations of activists. This pedagogical function was amplified through organisational replication. Inspired by the political models of groups such as GAPA and Pela Vidda, new AIDS NGOs were established across the country often adopting similar principles, organisational forms and even statutes (Contrera 2000; Parker 2020).

This is not to say that the AIDS movement was homogeneous or ideologically unified. On the contrary, the literature and interviews reveal tensions between what were often labelled ‘political NGOs’, that is, committed to structural transformation and rights‐based advocacy, and ‘charity NGOs’, which primarily offered services, such as hospice care, without a broader political agenda (Klein 2001; Rich 2019; Galvão 2000). However, in the early stages of the field's formation, these divergences did not pose a serious challenge to the prevailing orientation. As one informant put it, these differences ‘did not yet disturb the consensus’ (Interview 26).

In Bourdieusian terms, the early AIDS field was governed by a relatively stable doxa—a set of self‐evident truths that structured the space of possibilities. This doxa, rooted in solidarity, rights, and democratic participation, was naturalised through the practices of the dominant NGOs and reproduced in training, discourse and field alliances. The symbolic capital of key actors, drawn from their educational credentials, political histories, and media legitimacy, enabled them to monopolise the definition of legitimate participation.

The various achievements of the movement in the AIDS policy field served to reinforce and institutionalise the prevailing doxa. In 1985, the Ministry of Health created the National AIDS Programme, and one year later, the National AIDS Commission (CNAIDS), which formally included civil society organisations such as ABIA and the Gay Group of Bahia (Galvão 2000; Parker 2003). These institutional gains confirmed the legitimacy of the activist approach and marked a moment in which the field of power opened space for AIDS activists to intervene directly in policy formulation and to occupy positions of influence within the state apparatus.

Nevertheless, some scholars have questioned the extent to which the movement's universalistic conception of solidarity successfully mobilised society at large. As Terto (1999) notes, measuring the broader social resonance of this discourse is difficult. Furthermore, as Valle (2002) explored in his study, the dominant representation of the disease and of those affected by it in the 1980s and early 1990s was that of the *aidético* promoted by the journalistic field. The term had a strong symbolic effect as it was linked with decay, death, stigma and stigmatised groups such as homosexuals, who had had an ‘immoral and promiscuous trajectory’ (Valle 2002). However, the universalist discourse seemed to have informed, at least for some time, the activist fight (Valle 2002). With privileged access to major newspapers and media platforms, AIDS NGOs conducted national campaigns and published opinion pieces that framed the epidemic as a collective concern. Slogans such as ‘AIDS is everyone's problem’ and ‘Together we can win the fight against AIDS’ disseminated a vision of shared vulnerability and mutual responsibility (Bonfim 1987; Contrera 2000; Parker 2020).

From 1985 to 1989—the period of the programme's establishment and early expansion—the National AIDS Programme incorporated many of the principles articulated by the movement, particularly those developed by state‐level initiatives and NGOs (Parker 2003). This was also the period in which movement advocacy was at its most vibrant (Galvão 2002). The 1990s brought further victories, such as the Ministry of Health's decision to distribute AZT (zidovudine) freely in 1992 (Paiva 2003) and, even more significantly, the 1996 law guaranteeing universal access to triple therapy (Teixeira 2003). These milestones not only advanced the health outcomes of PLHIV but also reaffirmed the political capacity of the movement to shape national policy.

However, the political doxa, forged in a specific historical moment of democratic transition and social mobilisation, would not remain unchallenged. The next section explores how the demographic transformation of the epidemic—its pauperisation—introduced new actors with different life experiences, forms of capital and habitus into the field, unsettling the previously dominant logic of politicised activism.

## 3 The Pauperisation of the Epidemics

The pauperisation of AIDS in Brazil refers to a demographic shift in the epidemic's incidence, particularly the increase in cases among individuals with lower levels of formal education (Brito et al. 2001; Fonseca et al. 2000).^2^ Although early diagnoses in the 1980s primarily affected individuals with higher education levels, by 1999–2000, 74% of all newly reported AIDS cases were among individuals who were either illiterate or had completed only elementary school (Brito et al. 2001). Fonseca et al. (2000) found that in all Brazilian regions, the epidemic first affected more educated groups and progressively spread to less educated populations. This epidemiological shift would soon be mirrored in the composition of the AIDS movement itself.

The foundation of the National Network of PLHIV/AIDS (RNP+) in 1995—modelled on the Global Network of PLHIV (GNP+)—marked a significant moment in the field, seen as a culmination of the tension between ‘clinical identities’ versus the universalist ideology promoted by NGOs such as Pela Vidda (Valle 2002). RNP+ quickly became a ‘movement within the movement’ (Interview 14). By 2015, it counted approximately 1000 affiliates (Interview 46), and its membership reflected the demographic changes in the epidemic. A 2019 study found that the majority of RNP+ members had family incomes below two minimum wages, and only 16%–17% held a university degree in 2015 and 2017, respectively (Beloqui 2019). As a caveat, this is not to say that all members of the network are activists. As Biehl (2007) noted, the poorest AIDS patients rarely become activists.

A recurring theme in interviews with activists from long‐established ‘political’ NGOs is that the pauperisation of the epidemic was accompanied by a perceived impoverishment of the political debate about AIDS. Although the 1980s saw the consolidation of a dominant activist habitus—politicised, educated and connected to human rights discourses—many of the new entrants into the movement, themselves shaped by more precarious social conditions, brought a different set of dispositions into the field. As one prominent activist explained:

In the beginning […] [activists] were people who would come to Parliament [to do advocacy], who mastered the issue [of AIDS] and were able to have a major political discussion about AIDS […] who came from a socioeconomic and educational background that was well defined […] There were people who lived with HIV/ AIDS and who had come from academia, from the middle and upper classes, so there was less of an educational problem [because] the HIV had not migrated to the outskirts […].

(Interview 22)

Another activist explained how this shift affected formal spaces of participation such as CNAIDS and the National Health Council:

When the epidemic is marked by social determinants, and the people affected […] are those who do not have access to formal education and culture that provide a greater cognitive cloak, it ends up erasing the political discussions.

(Interview 42)

As Bourdieu (1984a) argues in *Distinction*, political preferences and capacities for abstract discussion are not simply individual choices but are conditioned by one's position in social space. Access to cultural capital—including formal education and exposure to institutional discourse—profoundly shapes one's ability to engage in political debate. In this context, the educational gap between older highly educated activists and newer participants became a barrier to continuity in the symbolic and political logics of the field. However, the lack of cultural capital is not the only contributor to the de‐politicisation of the movement. According to my informants, the pauperisation of AIDS meant the priority of the movement has also had to change as other needs and emergencies had to be addressed, such as lack of food, housing or any type of support necessary for treatment to succeed (Interview 22, Biehl 2007).

Rather than engaging in more ‘political’ debates which seek to symbolically transform AIDS activism, then, activists find themselves busy providing services that the state should provide (interviews 1, 19, 22, 37, 42 and 43). Political activism is rarely their priority. Indeed, Bourdieu (1984a) had noted that material necessities distance people from the pursuit of symbolic capital. According to an informant, ‘the networks of PLHIV became experts in distributing food parcels during COVID’ (Interview 15). Comparing their activities during the Covid‐19 pandemic with the work done by NGOs who produce research and analysis, one of my informants said ‘[certain NGOs produced] documents, documents, documents…but people do not eat documents’. In contrasting the two types of work, that is, political versus charity work, this extract exemplifies a broader recurring discourse from part of the movement: that for activists engaged in providing food parcels or services in general, sorting out the most urgent issues was what mattered the most.

In this context, those who are better equipped and positioned to deal directly with the dispossessed and marginalised are those who share their seropositivity, and often their life experiences, habitus and social position. Organisations formed exclusively by PLHIV according to a specific clinical conception of the term, that is, as those who have tested positive for HIV, started to emerge (Terto 1999; Valle 2002). According to Parker (2011), based on identity politics, this has been the most important division in the field which has prevented the formation of a broad‐based coalition to fight HIV/AIDS.

Following the creation of RNP+ in 1995, two other national networks were established: the National Movement of PositHIVe Female Citizens (MNCP) in 2004 and the Network of Adolescents and Young PLHIV/AIDS (RNAJVHA) in 2008. These networks reflected both the feminisation and rejuvenation of the epidemic and the restructuring of the activist field along new lines of identification and social experience.

In sum, pauperisation significantly altered the composition and dynamics of the field through the growing influence of networks and the precarious conditions of PLHIV. Although there is exchange between NGOs and networks—many activists belong to both—this internal division has produced competing forms of distinction. The result is a field marked by increasing fragmentation.

Although the changing composition of the activist field altered its internal dynamics, it is essential to understand how adjacent and broader structural shifts—particularly within the Brazilian AIDS policy field, the international development arena and broadly speaking the field of power—intensified these changes. The following section turns to these external transformations to analyse how technicisation, donor logics and the pharmaceuticalisation of public health reshaped the strategic orientations of AIDS activism in Brazil.

### 3.1 Broader Field Dynamics

#### 3.1.1 The Brazilian AIDS Policy Field and the Field of International Development

The pauperisation of the epidemic cannot account for all the changes in the AIDS movement. The dynamics of the broader social field where the AIDS movement operates, that is, the Brazilian AIDS policy field, have exerted considerable influence on the dynamics of the AIDS movement. In the 1990s and beginning of the 2000s, the National AIDS programme allocated a large amount of resources for NGOs to work on HIV/AIDS prevention programmes and advocacy (Rich 2019; Parker 2003; Barros and Silva 2016). This also enabled the AIDS movement to grow substantially from a few dozen organisations in 1998 to more than a thousand by 2010 (Rich 2019).

The changes in the Brazilian AIDS policy field were interwoven with another social context that had a direct effect on Brazil's National AIDS programme, that is, the field of international development with powerful actors such as the Ford and MacArthur foundations and International Financial Institutions (IFIs). AIDS and the developing world, including Brazil as one of the countries with the highest number of reported AIDS cases in the world (World Bank 2002), represented a prominent health agenda for donors and IFIs (Interview 2). It was mostly with large loans from the World Bank requiring the participation of civil society that the National AIDS programme funded NGOs through specific projects through open calls (Parker 2003; Rich 2019). These required a certain level of expertise or know‐how, not only for the application process but also for the management of the projects, its activities and budgeting (Interviews 3, 47). The new logic advanced by donors, which Galvão (1997) called ‘the dictatorship of projects’, meant NGOs needed to be staffed by people who possessed technical and professional capital (know‐how on how to write proposals), but not necessarily militant capital (Interview 3, 47). Often, these people did not live with HIV in the strict sense of the term, which caused grievances amongst those living with HIV, who did not feel represented (Interview 47). As noted, part of the literature argues that the ‘dictatorship of projects’ also contributed to reducing the contentious nature of AIDS NGOs (Galvão 1997; Parker 2003).

Influenced by the Denver Principles (UNAIDS 2023), whose slogan ‘nothing about us without us’ had become widespread amongst social movements in the 1990s, PLHIV/AIDS started voicing their frustration against the ‘technicisation’ of NGOs and communicating the idea that actions and policies aimed at them should have their direct participation (Interviews 46 and 47). The discourse that legitimised the establishment of the RNP+ criticised the bureaucratisation of NGOs and the change of their political objectives into ‘corporate interests, project management, with the purpose of guaranteeing their survival’ (Terto 1995, 11; see also Valle 2002).

The initial strategy of the activists who founded the RNP+ involved a ‘return to the sources’ (Bourdieu 1993) whereby activists criticised the neoliberalisation of the movement and used the same symbolic tools mobilised by dominant NGOs (in this case, the call for a politicised activism) to improve their position in the field (Interviews 43, 46 and 47).

However, I argue that what happens in the long‐term, partly due to the pauperisation of AIDS, partly due to a search for distinction and a challenge to—or ‘transgression’ (Bourdieu 1984a) of—the activist doxa, has contributed to rendering the movement less political. Within an overly populated field, networks of PLHIV sought to accrue symbolic capital in order to improve their positions. For this purpose, a new battle for the (re)definition of what it meant to exercise a legitimate form of activism, and with it, to change the doxa of the field, started. If the division between ‘political’ and ‘charity’ NGOs had existed since the 1980s, a more complex division gains strength in the 1990s and 2000s, based on other identities (see Valle 2015). Their strategy involved the redefinition of the main principles that had been established in the 1980s, particularly solidarity and the importance of being ‘political’. The symbolic competition around the label ‘AIDS activism’ became more intensified.

Rather than involving a collective battle for a broader socio‐political project that seeks to tackle the social determinants of health, my data shows that solidarity for activists from networks was often linked with support from ‘[activists] who actually know and see what people go through on the ground […] [who] experience AIDS, the everyday life [of those who live with HIV/AIDS]’ to those who live with HIV, who are starving and who suffer from prejudice in their day‐to‐day lives (Interview 25).

It is the specific rather than universal conception of PLHIV that characterises a legitimate activist who exercises solidarity ‘on the ground’ as opposed to an activism that takes place exclusively in the parliament or spaces of social representation in policymaking, such as CNAIDS, the National Commission of Articulation with Social Movements (CAMS in Portuguese), and the CNS. Furthermore, the signifier ‘political’ is no longer connected to ‘solidarity’, although the very justification for not doing political work is an acknowledgement that this still confers legitimacy to activism. As an informant from the Network of Women put it,

Great part of the women who arrive here want support and solidarity; they don’t want to discuss politics. Political discussions are great, I like them, but they don’t mobilize […] our women need to be heard, they need mutual help, [they need] to talk about their distress, the lack of money, the partner who beats them up […].

(Interview 10)

Talking about their activism, an informant from RNP+ stated:

I am not going to give a lecture on politics to those who are starving. Those who have AIDS and are hungry don't want to talk about ministerial directives […] The main pillars of RNP+'s practices are providing support [to PLHIV] and fighting for AIDS policy, [however] when these pillars were established, the vulnerability of PLHIV was not taken into consideration […] most of them are from an underprivileged social class.

(Interview 25)

It is important to note that these two activists were not asked if they did political work; in fact, the word ‘political’ was never mentioned. There was an embedded presupposition in these activists' habitus, thus, that what is expected from the AIDS movement is to do political work, that is, engage in political discussions that aim to raise awareness about social determinants of health and about policymaking more specifically. This shows, I suggest, that the idea of a politicised activism still carries legitimacy in the field.

Although acknowledging the history of, and achievements by, ‘powerful NGOs’ who do political work, these activists attempt, on the other hand, to differentiate themselves in a strategy to seek distinction. The following extracts further illustrate this attempt: ‘One thing is to work with HIV, another thing is to *live* with HIV. We are the protagonists of this story’ (Interview 25). For most, PLHIV have more legitimacy than ‘[t]he director of the NGO [who] does not know what I go through’ (Interview 11).

This new form of symbolic capital is mobilised to subvert the doxa. Solidarity becomes a more restricted concept that applies to the specific conception of PLHIV, and with a specific class, gender or age. It is more ethical than political in the sense that it concerns immediate, urgent issues that must be addressed rather than a more long‐lasting political project, although networks also engage in advocacy in parliament and policymaking in commissions.

It is difficult to know exactly to what extent these new ways of articulating activism in AIDS challenged the doxa of the field and its hierarchy. Bourdieu (1977), had argued that in times of crisis doxic beliefs are challenged as a result of a mismatch ‘between the subjective structures and the objective structures’. With pauperisation, the emergency required by the realities on the ground did not allow activists (who, furthermore, brought in different habitus and expectations) to talk about politics when ‘more urgent’ matters were at stake. To some extent what was doxic in the 1980s and 1990s was allocated to the discursive field, such as the discussion of whether politics mattered, and a new symbolic power came into being. This symbolic power could be observed in the way several activists who did not live with HIV excused themselves for being in a position of power within the movement—occupying the place of those who lived with HIV and who supposedly had more legitimacy to talk about AIDS.

It was also difficult to measure exactly how much these networks were able to translate this symbolic capital into economic resources. However, there is clear evidence that they have gained a more prominent position within the field of the AIDS movement and the field of AIDS policy. First, they gained visibility and political significance within ENONG, the National Meeting of NGOs (Lindner 2005, interview 45). Having a place at ENONG means organisations and networks can choose representatives for spaces of social participation in policymaking, such as the CNS, CAMS and CNAIDS.^3^ Networks increasingly occupied positions within these commissions, which according to my informants was itself a sign and source of symbolic power. Importantly, disputes for places within commissions started to be defined by the networks rather than by or within ENONGs. Finally, networks started to compete for and execute projects whose resources came either from the government or international donors (Communication MS). Their seminars, forums, projects and annual national and regional meetings received financial and technical support from local and national governments^4^—which many saw as an attempt to co‐opt and divide the movement.

If the loan from the World Bank encouraged the technicisation of NGOs, and later the establishment of networks, the loss of resources that the field experienced from the end of the 2000s exacerbated the struggle for distinction in—and the fragmentation of—the field (Interview 47). The withdrawal of funds from donors in the global North happened due to the Global Financial Crisis but also to the fact that Brazil was no longer a priority because it had been considered a case of success in fighting HIV/AIDS, thanks to universal distribution of triple therapy (Kenworthy et al. 2017; Interview 2, Parker et al. 2022).

By the end of the 2000s, the AIDS movement was fragmented, lacking economic capital and a consensus over what it meant to do legitimate activism. As activists noted, the participation of people who had different habitus and lacked militant capital was characterised by a lack of political engagement in the commissions where they would ‘sit in silence’ rather than positioning themselves politically to contest government decisions (Interviews 22 and 45). There are similar accounts of advocacy in parliament, as one activist describes ‘I do a lot of the advocacy by myself not only because we have great difficulty reaching a consensus, but also because we need political education within the social movement’ (Interview 21). Others describe advocacy today as attending to very specific—rather than collective—needs (Interview 22, 45). As one activist put it, ‘solidarity has become very specific…today it is everyman for himself!’ (Interview 45).

#### 3.1.2 The Field of Power

Both internal and external changes discussed above are intertwined with transformations in the ‘field of power’. For Bourdieu (2020), 33) the field of power is ‘[…] a space of positions from which power is exerted over species of capital that dominate in the different social fields that give access to this space […]’. Bourdieu (1998b) argued that neoliberal ideology reshaped the field of power, submitting every field, including that of international development and politics, to the laws of the market.

International Financial Institutions are key actors in the field of power. In the Global South, through structural adjustment programmes (SAP) the World Bank and the IMF promoted neoliberal reforms according to which states were restructured to withdraw from direct service provision, with NGOs stepping in to fill the gap (Kamat 2002). The ‘dictatorship of projects’ (Galvão 1997), as part of this logic, has rendered development (and activism) technical and depoliticised.

Big pharmaceutical corporations have also drawn on their economic might to reshape public health policy in a neoliberal fashion. With the invention of new and promising technologies of prevention and biomedical strategies (see Cohen 2010), Gilead Sciences, alongside powerful actors such as the Joint United Nations Programme on HIV/AIDS (UNAIDS) and the Global Fund, started promoting the idea that a cure for HIV/AIDS was possible and immanent (Kenworthy et al. 2017; Cueto and Lopes 2021). The ‘End of AIDS discourse’ promised a ‘magic bullet’ approach that has had two effects on the AIDS movement and on HIV policy in Brazil (see Parker et al. 2022), but also globally, according to Kenworthy et al. (2017): the tapering of donor support for NGOs to tackle more structural problems, which ended up being minimised or sidelined, and the adoption by governments of technical quick‐fix solutions rather than long‐term policies that focus on the social determinants of health (see Parker et al. 2022).

Particularly in developing countries which have significant socioeconomic issues, the ‘neoliberalisation of HIV prevention’ (Seffner and Parker 2016) has transformed patients in ‘homogeneous consumers of medicines’, minimising structural inequalities (Seffner and Parker 2016; Parker 2020; Parker et al. 2022) that have contributed to the pauperisation of AIDS.

In Brazil, this discourse found support in Dilma Rousseff's government (Parker 2020), more prominently from 2013 to 2016, reinforcing an increasingly pharmaceuticalised concept of public health (see Biehl 2007; Interview 1, 2, 34, Parker 2020; Parker et al. 2022) and the technicisation of the movement. According to activists from political NGOs, the main pillar of the national AIDS programme became, thus, a response based on biomedical technologies (Corrêa 2016; Parker 2020; interview 1, 2). State funding was redirected from political work towards a more technical type of work (see Corrêa 2016, Interview 9). NGOs found themselves competing for funds from the federal government to deliver services such as testing (Interview 9).

Neoliberalism and the pharmaceuticalisation of policy as part of its logic did not only render the lives of PLWHA more precarious, as extensively explored by Biehl's ethnographies (2007; 2009). AIDS NGOs have also been subjected to a form of institutional precarity. Informants from previously dominant NGOs pointed to the erosion of basic organisational resources—from technological infrastructure to physical office space—as evidence of the material effects of cuts, state policies and neoliberal funding frameworks which undermined their stability, continuity and legitimacy as collective actors (Interviews 1 and 2). As Butler (2009) defines it, precarity is not an individual condition but a socially, economically and politically produced vulnerability. In the field of the AIDS movement, this vulnerability translated into NGOs becoming fragile, competitive and increasingly incapable of sustaining contentious political action.

Neoliberalism has encouraged individual responsibility rather than collective and structural changes, which dominant AIDS NGOs had initially promoted. It is not far‐fetched to suggest that the very strategy used by networks in search for distinction reflects, inadvertently, the neoliberal trend. Critics have argued that identity politics, under neoliberalism, has shifted from challenging power structures to seeking recognition within them (Melamed 2006; Fraser 1997). The radical potential of identity politics can only be realised if connected to material and coalitional struggle aimed at structural change (Banet‐Weiser 2018; Taylor 2016). Instead, in the field of the AIDS movement there has been an emphasis on individual recognition over systemic transformation, diverting attention from collective struggles against structural inequalities.​

## 4 Conclusion

This article has argued that the depoliticisation of Brazil's AIDS movement is best understood as a complex process shaped by both external transformations in the field of power and internal restructuring within the field of activism. Drawing on Bourdieu's theory of practice, I have shown how symbolic struggles over the definition of legitimate activism together with material changes such as the pauperisation of the epidemics contributed to the weakening of a once highly politicised movement.

Rather than viewing demobilisation merely as institutional co‐optation or a loss of contentious politics, I have conceptualised depoliticisation as a field effect: the result of shifting distributions of capital and the entry of new agents with different habitus and life conditions. The early AIDS activists, whose dispositions had been shaped by prior engagement in democratisation and human rights struggles, had established a political doxa rooted in solidarity and structural transformation. Over time, however, this politicised vision was challenged by new actors whose life trajectories were marked more by precariousness than militancy, more by urgent survival needs than symbolic struggle. As the habitus of the field changed, so too did its stakes and logic of action.

The neoliberal transformation of the broader field of power intensified this process, reshaping the logics of funding, legitimacy and participation. NGOs once engaged in advocacy became service providers; collective rights were reframed as individual responsibilities and the collective struggle as individual fights for distinction (see Bourdieu 1998b). However, Bourdieu's concept of agency, understood as the practical capacity to act within and upon the field conditioned by one's habitus and capital, is never fully extinguished (Bourdieu 1990). Just as multiple crises have historically driven transformations within the fields of the AIDS movement and AIDS policy, new crises may likewise generate disruptions between activists' habitus and the evolving fields. The possibility of re‐politicisation involves renewed articulations of solidarity that seek to re‐anchor the struggle in collective, structural critique and transformation. Understanding the conditions of this potential reactivation, however, requires ongoing attention to the interplay between capital, habitus and field.

## Author Contributions

**Helena de Moraes Achcar:** conceptualization, investigation, funding acquisition, methodology, validation, writing – review and editing, formal analysis, project administration, data curation, resources, writing – original draft.

## Conflicts of Interest

The author declares no conflicts of interest.

## Data Availability

The data that support the findings of this study are available on request from the corresponding author. The data are not publicly available due to privacy or ethical restrictions.

## Endnotes

^1^
While some scholars reject the idea of a ‘field’ of social movements on the grounds of weak institutionalisation, the absence of a specific illusio, and specific capital accumulated for its own sake (Mathieu and Roussel 2012), I argue that the AIDS movement functions as a field insofar as it displays structured power relations, struggles over legitimacy, and relatively stable mechanisms of capital accumulation and conversion.

^2^
Education is used as a proxy variable for socioeconomic status. As a caveat, this does not mean, as Parker and Camargo (2000) had emphasised, that poverty (even when taken as a more complex category then solely education level) serves as a monocausal category for analysing vulnerability to HIV/AIDS.

^3^

http://antigo.aids.gov.br/sites/default/files/media/pagina/2016/59331_/informes_membros_cnaids_14maio_2013_pdf_16076.pdf (accessed 21 August).

^4^
See https://mncp.org.br/historia/ and https://www.rnpvha.org.br/quem‐somos/ (accessed 27 August 2024).

## References

[shil70187-bib-0001] Banet‐Weiser, S. 2018. Empowered: Popular Feminism and Popular Misogyny. Duke University Press. 10.2307/j.ctv11316rx.

[shil70187-bib-0002] Barros, S. G. 2018. Política Nacional de Aids: construção da resposta governamental à epidemia HIV/aids no Brasil. 1st ed. EDUFBA.

[shil70187-bib-0003] Barros, S. G. , and L. M. Silva . 2016. “A gênese da política de luta contra a aids e o Espaço Aids no Brasil (1981–1989).” Revista de Saúde Pública 50: 43. 10.1590/S1518-8787.2016050005801. 27463255 PMC4943519

[shil70187-bib-0004] Beloqui, J. 2019. “Brasil: violência e discriminação em pessoas vivendo com HIV/AIDS – a perspectiva dos membros da RNP+.” Relatório RNP+. https://giv.org.br/Arquivo/Relatorio‐RNP‐Brasil‐Violencia‐Discriminacao‐Pessoas‐HIV‐Aids.pdf.

[shil70187-bib-0005] Biehl, J. 2007. “Pharmaceuticalization: AIDS Treatment and Global Health Politics.” Anthropological Quarterly 80, no. 4: 1083–1126. 10.1353/anq.2007.0056.

[shil70187-bib-0006] Bonfim, P. C. 1987. “AIDS: um compromisso de todos nós.” Folha de São Paulo, de Novembro 29.

[shil70187-bib-0007] Bourdieu, P. 1977. Outline of a Theory of Practice. Cambridge University Press.

[shil70187-bib-0008] Bourdieu, P. 1984a. Distinction: A Social Critique of the Judgement of Taste. Routledge.

[shil70187-bib-0009] Bourdieu, P. 1984b. “Quelques Propriétés des champs.” In Questions de sociologie, edited by P. Bourdieu , 113–120. Éditions de Minuit.

[shil70187-bib-0010] Bourdieu, P. 1990. The Logic of Practice. Stanford University Press.

[shil70187-bib-0011] Bourdieu, P. 1993. Sociology in Question. Sage.

[shil70187-bib-0012] Bourdieu, P. 1998a. Practical Reason. Stanford University Press.

[shil70187-bib-0013] Bourdieu, P. 1998b. The Essence of Neoliberalism. Le Monde Diplomatique. https://mondediplo.com/1998/12/08bourdieu.

[shil70187-bib-0014] Bourdieu, P. 2005. “The Political Field, the Social Science Field, and the Journalistic Field.” In Bourdieu and the Journalistic Field, edited by R. Benson and E. Neveu , 29–47. Polity Press.

[shil70187-bib-0015] Bourdieu, P. 2020. “The Field of Power and the Division of the Labour of Domination.” In Researching Elites and Power, edited by F. Denord , M. Palme , and B. Réau , 31–52. Springer. 10.1007/978-3-030-45175-2_3.

[shil70187-bib-0016] Bourdieu, P. , and L. J. D. Wacquant . 1992. An Invitation to Reflexive Sociology. University of Chicago Press.

[shil70187-bib-0017] Brito, A. M. , E. A. Szwarcwald , and C. L. Landmann . 2001. “AIDS e infecção pelo HIV no Brasil: Uma epidemia multifacetada.” Revista da Sociedade Brasileira de Medicina Tropical 34, no. 2: 207–217. 10.1590/s0037-86822001000200010. 11391445

[shil70187-bib-0018] Buss, P. , and A. Pellegrini Filho . 2007. “A saúde e seus determinantes sociais.” Revista de Saúde Coletiva 17, no. 1: 77–93. 10.1590/s0103-73312007000100006.

[shil70187-bib-0019] Butler, J. 2009. Frames of War: When Is Life Grievable? Verso.

[shil70187-bib-0020] Camargo Júnior, R. 2003. “Prevenções de HIV/AIDS: desafios múltiplos.” Divulgação em Saúde para Debate 27: 70–80.

[shil70187-bib-0021] Cohen, J. 2010. “A Powerful and Perplexing New HIV Prevention Tool.” Science 330, no. 6009: 1298–1299. 10.1126/science.330.6009.1298. 21127220

[shil70187-bib-0022] Contrera, W. F. 2000. GAPAS: uma resposta comunitária à epidemia da AIDS no Brasil. Ministério da Saúde.” https://bvsms.saude.gov.br/bvs/publicacoes/179_2Gapas.pdf.

[shil70187-bib-0023] Corrêa, S. 2016. “A resposta brasileira ao HIV e à AIDS em tempos tormentosos e incertos.” In Mito vs realidade: sobre a resposta brasileira à epidemia de HIV e AIDS, edited by A. Bastos , R. Parker , and V. Terto, Jr. , 7–15. ABIA.

[shil70187-bib-0024] Crossley, N. 2003. “From Reproduction to Transformation: Social Movement Fields and the Radical Habitus.” Theory, Culture & Society 20, no. 6: 43–68. 10.1177/0263276403206003.

[shil70187-bib-0025] Cueto, M. , and G. Lopes . 2021. “Backlash in Global Health and the End of AIDS Exceptionalism in Brazil, 2007–2019.” Global Public Health 17, no. 6: 815–826. 10.1080/17441692.2021.1896764. 33689577

[shil70187-bib-0026] Daniel, H. 1994. Vida antes da morte. Rio de Janeiro: ABIA.

[shil70187-bib-0027] Davenport, C. 2014. How Social Movements Die: Repression and Demobilization of the Republic of New Africa. Cambridge University Press.

[shil70187-bib-0028] della Porta, D. , and M. Diani . 2020. Social Movements: An Introduction. 3rd ed. Wiley Blackwell.

[shil70187-bib-0029] Fonseca, E. M. 2007. “Descentralização, AIDS e redução de danos: a implementação de políticas públicas no Rio de Janeiro.” Cadernos de Saúde Pública 23, no. 9: 2113–2123. 10.1590/S0102-311X2007000900021. 17700948

[shil70187-bib-0030] Fonseca, M. G. , F. I. Bastos , and M. Passos . 2000. “A AIDS e grau de escolaridade no Brasil: Evolução temporal de 1986 a 1996.” Supplement, Cadernos de Saúde Pública 16, no. S1: 77–87. 10.1590/S0102-311X2000000700007. 10904391

[shil70187-bib-0031] Fraser, N. 1997. Justice Interruptus: Critical Reflections on the “Postsocialist” Condition. Routledge.

[shil70187-bib-0032] Galvão, J. 1997. “As respostas das ONGs brasileiras frente à epidemia de HIV/AIDS.” In Políticas, instituições e AIDS, edited by R. Parker , 69–108. Jorge Zahar/ABIA.

[shil70187-bib-0033] Galvão, J. 2000. AIDS no Brasil: a agenda de construção de uma epidemia. ABIA: Editora 34.

[shil70187-bib-0034] Galvão, J. 2002. 1980 a 2001: uma cronologia da epidemia de HIV/AIDS no Brasil e no mundo. ABIA.

[shil70187-bib-0035] Grangeiro, A. 2016. “Da estabilização à reemergência: os desafios para o enfrentamento da epidemia de HIV/AIDS no Brasil.” In Mito vs realidade. ABIA.

[shil70187-bib-0036] Grangeiro, A. , L. Laurindo da Silva , and P. R. Teixeira . 2009. “Resposta à aids no Brasil: contribuições dos movimentos sociais e da reforma sanitária.” Revista Panamericana de Salud Públic 26, no. 1: 87–94. 10.1590/s1020-49892009000700013. 19814887

[shil70187-bib-0037] Greco, D. 2016. “Trinta anos de enfrentamento à epidemia da AIDS no Brasil, 1985–2015.” Ciência & Saúde Coletiva 21, no. 5: 1553–1564. 10.1590/1413-81232015215.04402016. 27166903

[shil70187-bib-0038] Green, J. 2018. Revolucionário e gay: a vida extraordinária de Herbert Daniel. Civilização Brasileira.

[shil70187-bib-0039] Hardy, C. 2012. “Social Space.” In Pierre Bourdieu: Key Concepts, edited by M. em Grenfell . 2 ed., 229–249. Routledge.

[shil70187-bib-0040] Husu, H.‐M. 2013. “Bourdieu and Social Movements.” Social Movement Studies 12, no. 3: 264–279. 10.1080/14742837.2012.704174.

[shil70187-bib-0041] Kamat, S. 2002. Development Hegemony: NGOs and the state in India. Oxford University Press.

[shil70187-bib-0042] Kenworthy, N. , M. Thomann , and R. Parker . 2017. “From a Global Crisis to the “End of AIDS”: New Epidemics of Signification.” Global Public Health 13, no. 8: 960–971. 10.1080/17441692.2017.1365373. 28828943

[shil70187-bib-0043] Klein, C. , 2001. “A Associação Brasileira Interdisciplinar de AIDS.” In Solidariedade: A ABIA na virada do milênio, edited by R. Parker and V. Terto . ABIA.

[shil70187-bib-0044] Landau, C. 2011. “A Aids mudou de cara: Memória coletiva e novas oportunidades para o ativismo da AIDS no Brasil.” Plural 17, no. 2: 11–44. 10.11606/issn.2176-8099.pcso.2010.74538.

[shil70187-bib-0045] Landim, L. 1988. Sem fins lucrativos: Sociedade Civil e Organizações não Governamentais no Brasil. ISER.

[shil70187-bib-0046] Landim, L. 1993. Para além do mercado e do estado? Filantropia e cidadania no Brasil. ISER.

[shil70187-bib-0047] Lang, S. 1997. “The NGOization of Feminism.” In Transitions, Environments, Translations. Feminism in International Politics, edited by J. W. Scott , C. Kaplan , D. Keates , 101–120. Routledge.

[shil70187-bib-0048] Lapegna, P. 2013. “Social Movements and Patronage Politics: Processes of Demobilization and Dual Pressure.” Sociological Forum 28, no. 4: 842–863. 10.1111/socf.12059.

[shil70187-bib-0049] Lindner, L. 2005. ENONG ‐ Encontro Nacional de ONG/AIDS: Construção de sonhos e lutas. Lindner.

[shil70187-bib-0050] Malta, M. , and C. Beyrer . 2013. “The HIV Epidemic and Human Rights Violations in Brazil.” Journal of the International AIDS Society 16, no. 1: 18817. 10.7448/ias.16.1.18817. 24225350 PMC3827457

[shil70187-bib-0051] Mathieu, L. , and V. Roussel , eds. 2012. Penser les frontières sociales: Enquêtes sur la culture, l’engagement et la politique. Presses universitaires de Lyon.

[shil70187-bib-0052] Matonti, F. , and F. Poupeau . 2004. “Le capital militant.” Actes de la recherche en sciences sociales 155, no. 5: 4–11. 10.3917/arss.155.0004.

[shil70187-bib-0053] Melamed, J. 2006. “The Spirit of Neoliberalism: From Racial Liberalism to Neoliberal Multiculturalism.” Social Text 24, no. 4: 1–24. 10.1215/01642472-2006-009.

[shil70187-bib-0054] Ministério da Saúde . 2023. “Boletim epidemiológico: saúde da população negra.” 2. https://www.gov.br/saude.

[shil70187-bib-0055] Nunn, A. , S. Dickman , N. Nattrass , A. e Cornwall , and S. Gruskin . 2012. “The Impacts of AIDS Movements on the Policy Responses to HIV/AIDS in Brazil and South Africa: A Comparative Analysis.” Global Public Health 7, no. 10: 1031–1044. 10.1080/17441692.2012.736681. 23137055 PMC3738204

[shil70187-bib-0056] Paiva, V. 2003. “Beyond Magic Solutions: Prevention of HIV and AIDS as a Process of Psychosocial Emancipation.” Divulgação em Saúde para Debate 27: 192–203.

[shil70187-bib-0057] Parker, R. 1997. Políticas, instituições e AIDS: Enfrentando a epidemia no Brasil. ABIA/Jorge Zahar.

[shil70187-bib-0058] Parker, R. 2003. “Building the Foundations for the Response to HIV/AIDS in Brazil: The Development of HIV/AIDS Policy, 1982–1996.” Divulgação em Saúde para Debate 27: 143–183.

[shil70187-bib-0059] Parker, R. 2011. “Grassroots Activism, Civil Society Mobilization, and the Politics of the Global HIV/AIDS Epidemic.” Brown Journal of World Affairs 17, no. 2: 21–37. https://www.jstor.org/stable/24590789.

[shil70187-bib-0060] Parker, R. 2014. “A Cura da AIDS.” ABIAIDS. http://abiaids.org.br/?p=27249.

[shil70187-bib-0061] Parker, R. 2020. “AIDS Crisis and Brazil.” Oxford Research Encyclopedia of Politics. 10.1093/acrefore/9780199366439.013.865.

[shil70187-bib-0062] Parker, R. , M. Franch‐Gutiérrez , L. M. d. F. Silva , et al. 2022. “Políticas de HIV/Aids, ativismo e antropologia: conversando com Richard Parker.” Saúde em Debate 46, no. Número Especial 7: 277–289. 10.1590/0103-11042022e720.

[shil70187-bib-0063] Parker, R. , and K. R. Camargo, Jr. 2000. “Pobreza e HIV/AIDS: Aspectos antropológicos e sociológicos.” Supplement, Cadernos de Saúde Pública 16, no. S1: 89–102. 10.1590/s0102-311x2000000700008. 10904392

[shil70187-bib-0064] Rich, J. 2019. State‐Sponsored Activism: Bureaucrats and Social Movements in Democratic Brazil. Cambridge University Press. 10.1017/9781108626453.

[shil70187-bib-0065] Roth, S. 2019. “AIDS, Activism and Altruism.” Sociology 53, no. 2: 316–331. 10.1177/0038038518765577.

[shil70187-bib-0066] Roy, A. 2014. “The NGO‐nization of Resistance.” In Toward Freedom, 4. https://towardfreedom.org/story/archives/globalism/arundhati‐roy‐the‐ngo‐ization‐of‐resistance.

[shil70187-bib-0067] Seffner, F. , and R. Parker . 2016. “The Waste of Experience and Precariousness of Life: Contemporary Political Moment of the Brazilian Response to AIDS.” Interface: Comunicacao, Saude, Educacao 20, no. 57: 999–1010. 10.1590/1807-57622015.0827.

[shil70187-bib-0068] Taylor, K.‐Y. 2016. From #BlackLivesMatter to Black Liberation. Haymarket Books.

[shil70187-bib-0069] Teixeira, P. R. 2003. “Universal Access to AIDS Medicines: The Brazilian Experience.” Divulgação em Saúde para Debate 27: 184–191.

[shil70187-bib-0070] Terto, V., Jr. 1995. “Trocando idéias com Jorge Beloqui: donos da própria voz.” Boletim ABIA 30: 6–7.

[shil70187-bib-0071] Terto, V., Jr. 1999. “Seropositivity, Homosexuality and Identity Politics in Brazil.” Culture, Health and Sexuality 1, no. 4: 329–346. 10.1080/136910599300923. 12295532

[shil70187-bib-0072] Thomson, P. 2012. “Field.” In Pierre Bourdieu: Key Concepts, edited by M. Grenfell , 2nd ed. Routledge.

[shil70187-bib-0073] Thörn, H. , and S. Svenberg . 2016. “We Feel the Responsibility That You Shirk’: Movement Institutionalization, the Politics of Responsibility and the Case of the Swedish Environmental Movement.” Social Movement Studies 15, no. 6: 593–609. 10.1080/14742837.2016.1213162.

[shil70187-bib-0074] Tilly, C. , and S. Tarrow . 2015. Contentious Politics. Oxford University Press.

[shil70187-bib-0075] Uitermark, J. , and W. Nicholls . 2014. “From Politicization to Policing. The Rise and Decline of New Social Movements in Amsterdam and Paris.” Antipode 46, no. 4: 970–991. 10.1111/anti.12025.

[shil70187-bib-0076] UNAIDS . 2023. The Denver Principles: 40 Years on. https://www.unaids.org/en/resources/presscentre/featurestories/2023/june/20230626_denver‐principles‐40‐years‐on.

[shil70187-bib-0077] Valle, C. G. D. 2002. “Identidades, doença e organização social: um estudo das “Pessoas Vivendo com HIV e AIDS”.” Horizontes Antropológicos 8, no. 17: 179–210. 10.1590/s0104-71832002000100010.

[shil70187-bib-0078] Valle, C. G. D. 2015. “Biosocial Activism, Identities and Citizenship: Making up 'People Living With HIV and AIDS' in Brazil.” Vibrant: Virtual Brazilian Anthropology 12, no. 2: 27–70. 10.1590/1809-43412015v12n2p027.

[shil70187-bib-0079] World Bank . 2002. Stemming the HIV/AIDS Epidemic in Brazil. World Bank. https://documents1.worldbank.org/curated/en/974981468226770343/pdf/917340BRI020020c0Box0385351B0PUBLIC.pdf.

